# How perceiving pace of social change affects loneliness: psychological obsolescence as predictor for loneliness in late-life sample

**DOI:** 10.1007/s10433-026-00951-8

**Published:** 2026-09-17

**Authors:** Nora M. Degen, Frieder R. Lang

**Affiliations:** Institute of Psychogerontology, Friedrich-Alexander-University of Erlangen-Nuremberg, Nuremberg, Germany

**Keywords:** Loneliness, Psychological obsolescence, Social change, Longitudinal

## Abstract

A specific challenge of later life relates to the perceived pace of social change (i.e., psychological obsolescence), which may contribute to enhanced feelings of loneliness. In a 2-year longitudinal study with two measurement occasions (i.e., 2021 and 2023), data were collected from *N* = 818 adults aged 75 years and older (*M* = 80.99, SD = 4.07, range = 75–95 years). Participants were recruited from an urban area in southern Germany. A baseline-adjusted autoregressive path model was used to examine the concurrent association of psychological obsolescence with baseline loneliness and its prospective association with follow-up loneliness, accounting for baseline loneliness. Loneliness was assessed using the 6-item De Jong Gierveld Loneliness Scale and modeled as a manifest variable. Perceived obsolescence was significantly associated with baseline loneliness, with higher loneliness observed among individuals reporting greater psychological obsolescence. At follow-up, psychological obsolescence also showed a significant prospective association with loneliness after accounting for baseline loneliness. Over the two-year period, loneliness demonstrated substantial temporal stability. Among the covariates, being socially isolated and not being in a partnership were associated with higher follow-up loneliness. Overall, the findings suggest that, in later life, perceiving oneself as out of touch with the times is associated with higher loneliness both concurrently and prospectively, beyond the effects of prior loneliness and other established covariates.

**Supplementary Information:**

The online version contains supplementary material available at https://doi.org/10.1007/s10433-026-00951-8.

## Introduction

Staying in sync with the times despite social changes, including shifts in values, norms, and intergenerational expectations, may be especially challenging in later life. Consequently, older individuals may perceive a growing disconnect between themselves and contemporary social developments. In the following, we refer to the term *social change* in its broadest sense to encompass changes in social structures, institutions, values, social roles, relationships, and patterns of human interaction within a society. When perceived social demands exceed an individual’s perceived capacity to keep pace, feelings of loneliness may arise. In aging psychology, the phenomenon of feeling disconnected from social change is referred to as psychological obsolescence (Brandtstädter and Wentura 1994) and was found to be moderately correlated with loneliness (Kaspar 2004). Accordingly, the present study examines the cross-sectional association between psychological obsolescence and loneliness at baseline. In addition, it investigates whether psychological obsolescence is associated with loneliness two years later, after accounting for baseline loneliness and established risk factors for loneliness in later life (e.g., relationship status, health, financial situation).

### Loneliness: its predictors in old age

Loneliness is defined as a subjective and negative experience resulting from a discrepancy between desired and experienced levels of one’s social relations (Perlman and Peplau 1981). Individuals at any stage of life can experience feelings of loneliness. However, among older adults (80 years and older), there is evidence of high loneliness prevalence (Kaspar et al. 2023) and mixed evidence regarding age-related change, with some studies suggesting an increase in loneliness in old age (Graham et al. 2024), while others report relative stability across adulthood (Mund et al. 2020). Additionally, previous research suggests that individuals aged 75 and older who view aging as a period of social loss are at greater risk of persistent loneliness, as they may invest less in new relationships and find it increasingly difficult to overcome lonely states on their own (Huxhold and Henning 2023). Quantitative aspects of social relations (e.g., contact frequency; Luhmann and Hawkley 2016), as well as factors such as living alone, low income, low education, functional limitations, and poor (self-perceived) health have been associated with loneliness among older adults (Cohen-Mansfield et al. 2016; Dahlberg et al. 2022). Among the various risk factors, changes in marital relationships, such as getting divorced or becoming widowed, are strong contributors to loneliness in later life (Aartsen and Jylhä 2011; Buecker et al. 2021; Dykstra et al. 2005; Hsieh and Hawkley 2018; Utz et al. 2014).

### Psychological obsolescence and loneliness

Psychological obsolescence represents a perceived discrepancy between one’s personal characteristics (e.g., abilities, knowledge, beliefs, and attitudes) and contemporary demands. As society changes across domains such as new communication practices, evolving social norms and values, and the increasing digitalization of everyday life, individuals may perceive their environment as evolving faster than they can adapt. From a life-course perspective, age stratification theory (Riley 1971) emphasizes that individuals of different ages age concurrently within a society that is itself continuously changing. This implies that subjective experiences of obsolescence may not be solely a function of chronological age. Rather, they may emerge from the interaction between perceived social change, cohort-specific exposure, and role transitions across the life course. In later life, these dynamics may become particularly salient because accumulated beliefs, knowledge, and routines have been shaped by earlier social contexts. Psychological obsolescence reflects the experience of not being able to keep up with the velocity and pace of such changes, often leading to feelings of being outdated or irrelevant. When individuals perceive themselves disconnected from societal developments, their fundamental human need to belong (Baumeister and Leary 1995) is not met, which may lead to feelings of loneliness. However, psychological obsolescence may threaten not only the need for relatedness but also other basic psychological needs, such as competence and autonomy (Deci and Ryan 2008).

Very few studies are addressing this perception of keeping up with today’s pace of time. Individuals with lower perceived obsolescence tend to report greater life satisfaction (Wirth et al. 2024), whereas stronger perceived obsolescence was linked to diminished generative attitudes (Hösch et al. 2023), and to a modest decline in life satisfaction (Wirth et al. 2024). It was observed that men and women aged 70 and older tended to experience particularly high psychological obsolescence (Brandtstädter and Wentura 1994), especially after personal loss such as widowhood (de Paula Couto et al. 2025). Moreover, studies demonstrated that perceived obsolescence contributed to increases in late-life depression (Brandtstädter et al. 1997; Rothermund and Brandstädter 2003). Building on such findings, we suggest that perceiving oneself as out of touch with the times may undermine one’s social participation and connectedness. In this vein, we hypothesize that psychological obsolescence may represent an antecedent condition of loneliness in later life. Therefore, examining psychological obsolescence in late life may help identify individuals at risk of becoming or remaining lonely.

### Study aims and analytical procedures

The present study examined whether psychological obsolescence, defined as a perceived mismatch between the self and the experienced pace of social change, is associated with current and subsequent loneliness among individuals aged 75 years and older. To explore the temporal ordering of associations between psychological obsolescence and loneliness, we first estimated a manifest two-wave cross-lagged panel model (CLPM) including autoregressive paths for both constructs and adjusting for covariates. This model was used as a preliminary and descriptive analysis prior to fitting the primary structural equation model (SEM). To investigate cross-sectional and longitudinal associations with loneliness, a baseline-adjusted autoregressive path model was then estimated using an SEM framework, with psychological obsolescence specified as a predictor of follow-up loneliness while controlling for baseline loneliness and covariates. Loneliness at T1 was predicted by psychological obsolescence and covariates age, gender, relationship status, financial situation, education, subjective health, number of diagnoses, and social isolation. Loneliness at T2 was predicted by T1 loneliness (autoregression), psychological obsolescence, and the same covariates. This design allows for an assessment of how loneliness is associated with psychological obsolescence over time, providing a longitudinal perspective on predictors of loneliness while accounting for baseline loneliness.

We expected higher initial psychological obsolescence to be significantly associated with higher loneliness at follow-up, controlling for baseline loneliness. Analyses were performed in R version 4.5.2, using the “psych” package for reliability (Revelle 2011) and “lavaan” package for path modeling (Rosseel 2012).

## Method

### Sample and procedure

**Fig. 1:**
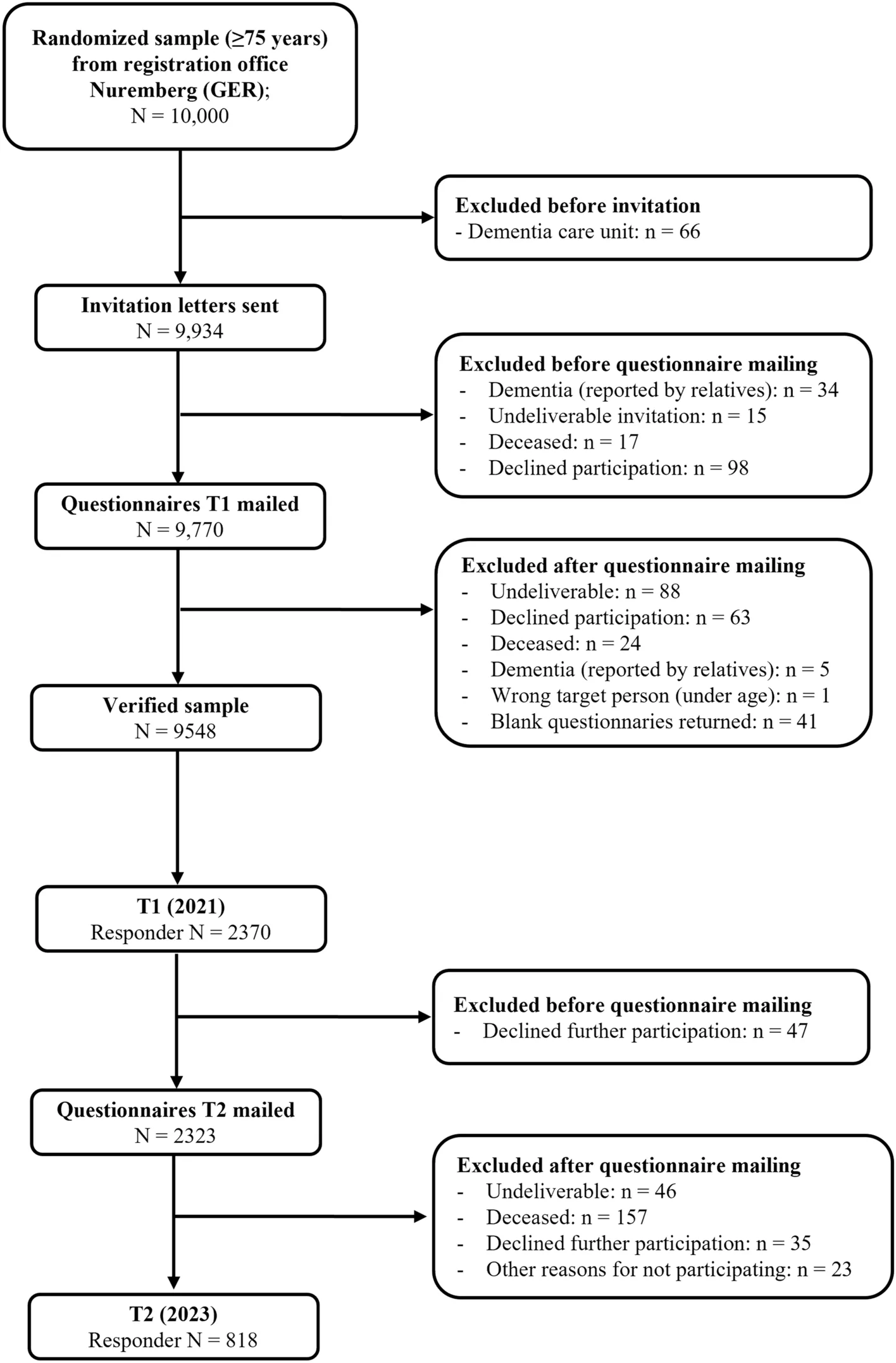
Flowchart of participant recruitment, study participation, and attrition across T1 and T2. Participants were provided with a paper questionnaire and an optional online version. Responses from the online mode (*n* = 169) at T1 are included in the total sample of responder at T2 (i.e., *N* = 2370). For T2, only paper-based questionnaires were used; online participation was no longer available

The study used German longitudinal data sourced from an ongoing Health and Social Participation Survey (Gesundheit und soziale Teilhabe, GesTe), collected in 2021 (T1) and 2023 (T2). The study was reviewed and approved in terms of compliance with ethical principles and data protection regulations (Ethical Commission of Friedrich-Alexander-University Erlangen-Nuremberg, Ethic Votum No. 23-237-S). The survey provides information on mental health, physical health, and social embeddedness of participants aged 75 and older. The sample was drawn from the population register of Nuremberg in 2021 using a randomized sample of 10,000 individuals. All participants received an invitation letter with the option to complete the questionnaire online via QR code. Participants could choose to opt out of participation via email, phone, or mail. If they did not decline, they were subsequently mailed a paper-and-pencil version of the questionnaire. The response rate for participation via the online survey was low (*n* = 169). Of the 9,548 eligible individuals in the verified sample, 2,370 (*M* = 81.76 years, SD = 4.53 years, range = 75–101 years; 54.6% female) returned a valid questionnaire (T1), corresponding to a response rate of 24.8%. For a detailed overview of the study flow and participant inclusion, see Fig. 1. Data collection for the second wave was conducted exclusively using mailed questionnaires, which participants completed at home and returned by post. Analyses for the current study were based on 818 longitudinal respondents, using full-information maximum likelihood (FIML) to account for missing data on study variables.^1^ For a detailed overview of the baseline characteristics of the cross-sectional, longitudinal, and attrition samples, see Table 1. To examine potential attrition bias, we conducted a logistic regression predicting continued participation at T2 (0 = dropout, 1 = retained). Results indicated that age (OR = 0.96, 95% CI [0.94, 0.99], *p* = 0.002) and social isolation (OR = 0.81, 95% CI [0.72, 0.91], *p* < 0.001) were negatively associated with continued participation. In contrast, higher education (vs. low education; OR = 1.38, 95% CI [1.01, 1.88], *p* = 0.04), being separated or single (vs. partnered; (OR = 1.44, 95% CI [1.03, 1.99], *p* = 0.03), better financial situation (OR = 1.16, 95% CI [1.03, 1.30], *p* = 0.01), and better subjective health (OR = 1.01, 95% CI [1.00, 1.01], *p* < 0.001) were positively associated with continued participation. These findings suggest that older participants and those with higher social isolation were less likely to remain in the study. Conversely, participants with higher education, those who were single, those who reported better financial resources, and those with better subjective health were more likely to remain in the study. Overall, the findings indicate modest selective attrition, with generally small effects.

**Table 1:** Baseline characteristics of the cross-sectional, longitudinal, and attrition samples

	Cross-sectional sample	Longitudinal sample	Attrition sample
Variable	M (SD) / %	M (SD) / %	
Loneliness T1	2.52 (2.71)	2.23 (2.55)	2.66 (2.78)
Loneliness T2	–	2.41 (2.56)	–
Age T1	81.76 (4.53)	80.99 (4.07)	82.17 (4.71)
Gender (*f*) T1	54.6%	53.5%	55.1%
*Relationship status T1*			
Partnered/married	57.03%	58.87%	54.46%
Single/separated	10.23%	11.99%	9.3%
Widowed	33.74%	29.13%	36.28%
Financial situation T1	4.17 (0.89)	4.28 (0.85)	4.12 (0.91)
*ISCED T1*			
Low	17.55%	16.26%	18.23%
Medium	48.69%	44.74%	50.77%
High	29.66%	36.06%	26.29%
Subj. health T1	66.55 (20.94)	70.49 (18.36)	64.42 (21.93)
Diagnoses T1	4.71 (2.52)	4.52 (2.31)	4.81 (2.61)
Social Isolation T1	0.62 (0.89)	0.45 (0.76)	0.71 (0.93)
Obsolescence T1	2.33 (0.97)	2.25 (0.95)	2.38 (0.98)

The cross-sectional sample includes all participants who completed the first assessment (*N* = 2370). The longitudinal sample includes participants who completed both assessments (*N* = 818) and constitutes the study sample analyzed in the present study. Participants who did not participate at T2 (*N* = 1552) were classified as attrition cases. Means and standard deviations are based on all available data; thus, the number of participants may vary across variables due to missing data. Subj. Health = Subjective Health. *M* and SD are used to represent mean and standard deviation, respectively

### Measures

#### Multi-item measurement of loneliness

The 6-item De Jong Gierveld Loneliness Scale (de Jong Gierveld and Van Tilburg 2010) was used to measure feelings of loneliness at T1 and T2. For negatively formulated items, responses were scored on a scale from 0 to 2 (0 = *no*, 1 = *more or less*, 2 = *yes*), whereas for positively formulated items, the scoring was reversed to maintain consistency in the direction of the total score. The result is an overall loneliness score ranging from 0 to 12, with higher scores indicating higher loneliness. The calculation of the score used here has been modified to the original index (0–6, with a cutoff of ≥ 2 as lonely) proposed by de Jong Gierveld and Van Tilburg (2010). The modified calculation (0–12) was used in previous studies to provide a more nuanced assessment of loneliness (e.g., Hansen and Slagsvold 2016). McDonald’s omega was computed using a two-factor model (Flora 2020) to account for the underlying dimensions (i.e., emotional and social loneliness). The resulting reliability coefficient was *ω* = 0.84 at both T1 and T2, indicating good reliability of the measurement instrument. To compare prevalence rates with other German panel datasets (i.e., German Ageing Survey; Socio-Economic Panel), the outcome sum score was dichotomized using a mean split, consistent with previous studies (e.g., Huxhold and Tesch-Römer 2021). This cutoff score identifies only very severe loneliness. Consequently, it identifies only a relatively small proportion of older adults as lonely.

#### Psychological obsolescence

To measure psychological obsolescence at T1, the following three items by Brandtstädter and Wentura (1994) were used: “*I have increasingly less sympathy for the views of the younger generation,”* *“I increasingly feel that I have lost touch with today’s time,”* and *“It is becoming increasingly difficult for me to deal with today’s way of life,”* with a 5-point rating scale where 1 is defined as *does not apply at all* and 5 as *does apply very much*. McDonald’s omega was *ω* = 0.76, indicating that the measurement instrument demonstrates satisfactory reliability.

##### Covariates

Sociodemographic variables included age, gender (0 = *female*, 1 = *male*), relationship status (1 = *married/partnered* [reference category], 2 = *separated/single*, 3 = *widowed*), education (based on ISCED; levels 0–2 = *low education* [reference category], levels 3–4 = *medium education*, levels 5–8 = *high education*), and financial situation (1 = “*I have to limit my spending a lot”* to 5 = “*I manage very well with my budget*”). To obtain an indicator of social isolation, respondents were asked three questions: how often they meet with a fixed group of people, how often they meet with individual friends, acquaintances, and relatives, and how often they talk to friends, acquaintances, and relatives on the phone or online. A similar scale for measuring social isolation was used by Emerson et al. (2021). The response options were 1 = *never*, 2 = *less frequently*, 3 = *1–3 times per month*, 4 = *once per week*, 5 = *several times per week*, and 6 = *daily*. For each of the three items, responses of *never* or *less frequently* (i.e., 1 or 2) were coded as indicating social isolation (1), whereas all other responses were coded as not socially isolated (0). The dichotomized items were then summed to create a social isolation score ranging from 0 to 3, with higher scores indicating greater social isolation. Self-rated health status was measured by a single item: “*please choose one point in this 0–100 scale, which can best represent your health today (0 means the worst and 100 means the best)*.” Indicators of objective health were surveyed with a sum composite score of indicators assessing 12 medical diagnoses and complaints (e.g., cardiovascular diseases, sleep disorders, hearing impairment, gastrointestinal diseases). This resulted in an index ranging from 0 to 12, with higher scores indicating more medical diagnoses and complaints. Similar checklist for physical health has been used by Böger and Huxhold (2018). Covariates were assessed at T1 (2021).

## Results

Table 2 presents the correlation matrices among the study variables. On average, loneliness was *M* = 2.23 (SD = 2.55; T1) and *M* = 2.41 (SD = 2.56; T2), indicating a significant mean increase (*p* = 0.04) in loneliness over time. When the loneliness score was used dichotomously, the prevalence in the sample was 8.64% at T1 (2021) and 8.29% at T2 (2023). This prevalence is consistent with those reported in other German panel studies (e.g., Wurm et al. 2023, 7.6%; Schobin et al. 2024, 10.2%). Psychological obsolescence was relatively low, with a mean of 2.25 (SD = 0.95) on a scale ranging from 1 to 5. Across the two timepoints, loneliness was showing small-to-moderate positive associations with psychological obsolescence (T1: *r* =0.29; T2: *r* = 0.26), suggesting that higher scores on loneliness tended to be associated with higher scores on psychological obsolescence. Moreover, loneliness was negatively associated with gender, relationship status, financial situation, education, and subjective health. It was positively associated with the number of diagnoses and social isolation. This suggests that men, individuals in relationships, and those with better financial situation, higher education, and better self-reported health tended to report lower loneliness scores. In contrast, individuals with more diagnoses and individuals engaging less in social activities (i.e., being socially isolated) tended to report higher loneliness scores.

**Table 2:** Correlations among study variables (*N* = 818)

Variable	*M/%*	SD	1	2	3	4	5	6	7	8	9	10
1. Loneliness T1	2.23	2.55										
2. Age T1	80.99	4.07	− .02									
			[− .08, .06]									
3. Gender (f) T1	53.5%		− .08*	.06								
			[− .15, − .01]	[− .01, .13]								
4. Relationship T1	0.59	0.49	− .16**	− .15**	.37**							
			[− .22, − .09]	[− .22, − .09]	[.31, .43]							
5. Financial Sit. T1	4.28	0.85	− .23**	.03	.03	.12**						
			[− .29, − .16]	[− .04, .09]	[− .04, .10]	[.05, .19]						
6. ISCED T1	2.20	0.71	− .10**	− .07*	.44**	.23**	.04					
			[− .17, -− .03]	[− .14, − .00]	[.38, .50]	[.16, .29]	[− .03, .11]					
7. Subj. Health T1	70.49	18.36	− .15**	− .14**	.01	.05	.18**	.03				
			[− .22, − .08]	[− .21, − .07]	[− .06, .08]	[− .02, .12]	[.11, .24]	[− .04, .10]				
8. Diagnoses T1	4.52	2.31	.13**	.14**	.02	− .05	− .16**	− .05	− .41**			
			[.06, .20]	[.07, .20]	[− .05, .09]	[-.12, .02]	[− .23, − .10]	[− .12, .02]	[− .46, − .35]			
9. Social isolation T1	0.45	0.76	.30**	.10**	.08*	.00	− .13**	− .06	− .17**	.08*		
			[.23, .36]	[.03, .17]	[.01, .14]	[− .06, .07]	[− .20, − .06]	[− .13, .01]	[− .24, − .10]	[.01, .14]		
10. Obsolescence T1	2.25	0.95	.29**	.14**	.00	.04	− .13**	− .10**	− .23**	.17**	.16**	
			[.22, .35]	[.07, .20]	[− .07, .07]	[− .03, .10]	[− .19, − .06]	[− .16, − .03]	[− .29, − .16]	[.10, .23]	[.09, .22]	
11. Loneliness T2	2.41	2.56	.67**	.03	− .09*	− .16**	− .22**	− .08*	− .13**	.13**	.26**	.26**
			[.63, .71]	[− .04, .10]	[− .16, − .02]	[− .23, − .09]	[− .29, − .15]	[− .15, − .01]	[− .20, − .06]	[.06, .20]	[.19, .33]	[.20, .33]

For the correlation analyses, relationship status coded as 0 = not partnered (includes single, divorced, or widowed), 1 = partnered. In the autoregressive model, relationship status was modeled using three categories (partnered, single/divorced, widowed). Financial Sit. = financial situation; Subj. Health = subjective health

^*^indicates *p* < 0.05. ** indicates *p* < 0.01

The cross-lagged analyses showed an association between psychological obsolescence at T1 and loneliness at T2, whereas the reverse association (loneliness at T1 with psychological obsolescence at T2) was not statistically significant (see Supplementary Table S1). We interpret these as exploratory and not as evidence for directional effects because the main focus of our present research was on predicting subsequent loneliness. Furthermore, we are not aware of any prior research addressing the change or stability in psychological obsolescence, so that we had no plausible hypothesis in this regard. The following in-depth analyses therefore focus on loneliness as the outcome within a theoretically specified baseline-adjusted SEM.

### Cross-sectional predictors of loneliness at T1

As shown in Table 3, there was a significant positive association between psychological obsolescence and loneliness at T1 (*β* = 0.238, *p* < 0.001). Individuals with higher scores in psychological obsolescence thus reported higher baseline loneliness. Additional risk factors of loneliness were being single/divorced (*β* = 0.167, *p* < 0.001), being widowed (*β* = 0.094, *p* < 0.01; reference group for both: married/partnered individuals), and being socially isolated (*β* = 0.243, *p* < 0.001). Higher age (*β* = − 0.096, *p* < 0.01) and a better financial situation (*β* = − 0.122, *p* < 0.001) were significantly associated with lower loneliness scores, indicating that both variables functioned as protective factors. Subjective health, number of diagnoses, education, and gender showed no significant association with baseline loneliness (*ps* > 0.05). The full model explained 22.3% of the variance in loneliness at T1, with psychological obsolescence accounting for an additional 5.2% of the variance beyond the covariates (Δ*R*^2^ = 0.052).

**Table 3:** Baseline-adjusted autoregressive path model predicting loneliness at baseline and follow-up (*N* = 818)

Outcome	Predictor	*β*	*B*	SE(*B*)	*p*
Loneliness T1	Age T1	− .096	− 0.060	.021	0.004
	Gender (*f*=0) T1	− .046	− 0.234	.195	0.229
	Relationship status (ref: partnered) T1				
	Single/divorced	.167	1.316	.265	0.000
	Widowed	.094	0.530	.202	0.009
	Financial situation T1	− .122	− 0.366	.100	0.000
	ISCED (ref. low education) T1				
	Middle education	− .040	− 0.205	.238	0.389
	High education	− .026	− 0.137	.267	0.608
	Subjective health T1	− .022	− 0.003	.005	0.534
	Diagnoses T1	.065	0.072	.039	0.062
	Social isolation T1	.243	0.814	.109	0.000
	Obsolescence T1	.238	0.644	.089	0.000
Loneliness T2	Loneliness T1	.601	0.602	.030	0.000
	Age T1	.026	0.016	.018	0.368
	Gender (*f*=0) T1	− .027	− 0.141	.165	0.394
	Relationship status (ref: partnered) T1				
	Single/divorced	.100	0.789	.228	0.001
	Widowed	.019	0.105	.173	0.545
	Financial situation T1	− .036	− 0.107	.086	0.217
	ISCED (ref. low education) T1				
	Middle education	.031	0.161	.205	0.431
	High education	.032	0.168	.229	0.463
	Subjective health T1	.014	0.002	.004	0.646
	Diagnoses T1	.028	0.031	.033	0.344
	Social isolation T1	.063	0.213	.099	0.031
	Obsolescence T1	.074	0.202	.079	0.011

All covariates were measured at baseline (T1)

### Longitudinal predictors of loneliness at T2

Loneliness at T1 significantly predicted subsequent loneliness (*β* = 0.601, *p* < 0.001), indicating temporal stability. Beyond this stability, psychological obsolescence was significantly associated with loneliness at T2 (*β* = 0.074, *p* < 0.05). This association suggests that psychological obsolescence is associated with higher loneliness over time, beyond prior loneliness.^2^ Among the covariates, the analysis showed significant positive associations for being single/divorced (*β* = 0.100, *p* < 0.01; reference group: married/partnered individuals) and being socially isolated (*β* = 0.063, *p* < 0.05), whereas financial situation and being widowed (ref. group: married/partnered individuals) were not significantly associated with loneliness at T2 (*ps* > 0.05). The model explained 47.3% of the variance in loneliness at T2, with psychological obsolescence accounting for an additional 0.5% of the variance beyond loneliness at T1 and covariates (Δ*R*^2^ = 0.005).

### Sensitivity analyses

As a sensitivity check, we re-estimated the primary model using only participants with complete data on all study variables. The complete-case analysis yielded results that were consistent with those of the primary model (see Supplementary Materials, Table S2), with no meaningful differences in the direction or interpretation of effects.

Because the specified lavaan model was fully saturated (df = 0), no global model test and, thus, no model-based power estimation within the SEM framework was possible. To evaluate the sensitivity of the study with respect to the effect of obsolescence, the corresponding regression equations were therefore considered separately. Based on the observed standardized regression coefficients, sensitivity analyses were conducted using G*Power (Faul et al. 2007). The results suggested that a sample size of *N* = 228 (power of 0.80 at an alpha level of 0.05) would be sufficient to detect small-to-medium effects of obsolescence. Variance inflation factors (VIFs) in the regression models did not exceed 1.5.

## Discussion

In the present research, we examined the cross-sectional and longitudinal associations between psychological obsolescence and loneliness in a two-year study of older adults aged 75 years and older. As expected, there were weak to moderate cross-sectional and longitudinal positive associations between perceived obsolescence and feelings of loneliness. Perceiving difficulty in keeping up with perceived demands of contemporary society was associated not only with interindividual differences in baseline loneliness, but also with higher loneliness at the two-year follow-up. Furthermore, social isolation and not being in a partnership were consistently associated with higher mean loneliness. Overall, findings indicate that psychological obsolescence is associated with loneliness over time, above and beyond other known correlates and baseline differences in loneliness.

### Psychological obsolescence in relation to perceived social exclusion

The finding that psychological obsolescence is associated with higher loneliness at the follow-up suggests that perceiving difficulty in keeping pace with the demands and the changes in one’s social environment may be relevant for understanding social disconnection in later life. One possible explanation for these findings is that psychological obsolescence reflects perceived social exclusion as proposed in the work of Bude and Lantermann (2006). However, we submit that perceived obsolescence and perceived social exclusion may reflect conceptually different social experiences. For example, a key aspect of perceived exclusion pertains to one’s own social position within society, specifically whether one perceived oneself as an accepted member of society (i.e., *am I part of society?*). In contrast, psychological obsolescence reflects feeling overwhelmed with demands resulting from changes and from experiencing a disconnection from younger generations (Hösch et al. 2023). Thus, it pertains to an experience of personal deficits in one’s capacity to participate in the society (i.e., *do I still fit in with the times?*). In line with Huxhold et al. (2022b), one may ask whether psychological obsolescence like perceived social exclusion and feelings of loneliness can be considered separate entities. Alternatively, we suggest that the perception of being unable to keep pace with the speed of social change may reflect a subjective experience of social alienation—as a component of loneliness specific to the aging process.

### Psychological obsolescence in the context of aging and social change

Furthermore, later life is often marked by discontinuities that require adaptational effort, so that the pace and degree of change are experienced as congruent with individual preferences and social demands (Atchley 1989). Older individuals may use their internal structure (e.g., experiences, skills) to adapt to their current situation (Nikitin and Freund 2019). Due to the COVID-19 pandemic and associated isolation restrictions, many aspects of everyday life shifted online, including social communication and daily tasks (Vargo et al. 2021). This placed older adults without internet access or sufficient digital literacy at greater risk of social and digital exclusion (Seifert 2020; Seifert et al. 2021). Consistent with this notion, virtual social contact was associated with lower loneliness among young and middle-aged adults, whereas among older adults, only face-to-face interactions were linked to reduced loneliness, underscoring generational differences in preferred forms of social contact (Zheng et al. 2026). As modern society continues to evolve, engaging with new technologies and novel forms of social and intergenerational exchange may require increasing coping efforts, particularly for individuals who find it challenging to adapt to these changes (Seifert 2023). For instance, psychological obsolescence has been associated with lower technology use and acceptance among older adults (Schmidt and Wahl 2019). While younger people may also experience a sense of obsolescence (e.g., feeling overwhelmed by technological innovations or unable to keep up with new trends), this experience may be more temporary and reversible as they may be more inclined to adopt new technologies (Hülür and Macdonald 2020). In contrast, for older individuals, psychological obsolescence may pose a threat to identity and self-continuity (Rutt and Löckenhoff 2016). Previous research has shown that lower self-continuity is associated with greater loneliness in later life (Lampraki et al. 2019). Given that the present study showed that psychological obsolescence is associated with higher loneliness, it may similarly have implications for individuals’ sense of self-continuity.

The participants in the present study belonged to birth cohorts spanning 1926–1946, born before and during World War II in Germany. Across their lifespan, these cohorts witnessed profound technological and social transformations (e.g., digitalization, new family forms), each requiring continuous adjustment to changing social conditions, which may increase the risk for perceiving enhanced obsolescence. Existing theoretical models of loneliness and social relationships in later life explain loneliness through age-related changes in social opportunities and resource investments (Differential Investment of Resources Model; Huxhold et al. 2022a), changes in the composition and support functions of social networks shaped by personal and situational characteristics (Social Convoy Model; Antonucci et al. 2014), and the fulfillment of social relationship expectations shaped by motives, contextual factors, and coping strategies (Social Expectation Framework; Akther-Khan et al. 2023). We suggest that perceived obsolescence in later adulthood may represent an additional factor that influences the evaluation of social relationships beyond the mechanisms proposed in these models.

### (Longitudinal) correlates of loneliness

The present findings align with previous research identifying similar factors for loneliness in later life. Perceived financial sufficiency was closely linked to lower loneliness at T1, consistent with other empirical findings (e.g., Cohen-Mansfield et al. 2009; Victor et al. 2021). In terms of relationship status, the present study showed that, compared to individuals in relationships, being single or widowed had a situational influence on increased feelings of loneliness, whereby being single also had a longitudinal impact. This contrasts with the prevailing findings, where widowhood is linked to an increased risk of loneliness (Vedder et al. 2024). However, it should be noted that we did not take into account the duration of relationship status, so widowed individuals may have adapted or secured sufficient social support from family or friends in the transition, whereas this process might be more challenging for single or divorced individuals, for whom the social circumstances remained the same. Consistent with prior work, loneliness and social isolation were moderately associated in the present study, supporting the view that they are related yet distinct constructs (e.g., Dahlberg et al. 2024). Moreover, our findings align with the notion that being socially isolated serves as a longitudinal risk factor for subsequent loneliness (Dahlberg et al. 2022).

Furthermore, psychological obsolescence showed only a weak association with social isolation. This suggests that associations of perceived obsolescence with loneliness cannot be reduced to a possible lack of contact. Instead, they may reflect a broader perception of no longer fitting into or being valued by contemporary society, encompassing not only reduced connectedness but also threats to autonomy and competence. In this vein, we did not find any mediation or moderation of social isolation on the association of perceived obsolescence with subsequent loneliness.

## Strengths and limitations

The present study design offers several strengths. By incorporating both cross-sectional and longitudinal components, this approach enables the examination of associations between predictors and loneliness at baseline, as well as their associations with loneliness at follow-up after controlling for baseline loneliness. This two-wave panel study also provides valuable information about the stability of loneliness during and after the Corona pandemic, shedding light on whether individual differences persist or shift within the study period. The paper-and-pencil design mitigated the potential risk of attrition due to a lack of online access among older individuals. Nevertheless, there are some limitations that need to be addressed. First, all study participants lived in an urban area in Germany, and this may lead to limited generalizability. Second, because the study started in mid-2021 during the pandemic, no information on participants’ pre-COVID-19 loneliness was available. However, as other studies have shown (e.g., Luchetti et al. 2020), older adults reported increased feelings of loneliness during the acute phase of the pandemic. The initial level of loneliness in the acute phase in 2020 may have been heightened and then decreased again, which is why individuals reported lower loneliness after more than a year of the pandemic. Considering the fact that lonely individuals are more likely to be under-represented in surveys (Pinquart and Sorensen 2001), follow-up participants appeared less lonely on average; however, loneliness did not significantly predict the likelihood of participation at T2. Third, the study did not take into account possible changes in sociodemographic (e.g., relationship status) and health factors (e.g., subjective health). However, these factors may have led to vulnerabilities that could have influenced the extent of loneliness at the second timepoint. Fourth, previous studies (e.g., Buecker et al. 2020) have shown that personality traits play a role in accounting for individual differences in loneliness, incorporating them in future studies may therefore enhance the proportion of explained variances. For instance, individuals who are more open to new experiences may also be more open to using new communication technologies to connect with others and thus enhance their sense of belonging. When it comes to the use of technology, components of technological motivation are also crucial in determining whether or not the technology is applied (Kamin and Lang 2013).

## Implications and future research

The present study indicates that perceiving oneself *in sync* with social change and today’s way of living can be a protective factor against loneliness. In contrast, many demands of modern life may result in feeling lonelier. Meta-analyses showed that interventions to reduce loneliness were most effective when they consisted of psychological interventions that addressed cognition or attention (Lasgaard et al. 2025; Masi et al. 2011). Therefore, psychological obsolescence as a cognitive representation of the present time should be examined for sensitivity to changeability in future studies. Furthermore, it remains unclear whether perceived obsolescence is related to self-continuity and to what extent perceptions of obsolescence are malleable for functioning, i.e., whether it affects the same outcomes of loneliness (e.g., depression). Although the subjective experience of not being able to keep up with the times may be more pronounced in older age, but it could also be a facet of loneliness in younger years. Studies using life-span samples are needed to clarify the extent to which this experience is age-specific or reflects loneliness across adulthood. Psychological obsolescence explained only a modest proportion of additional variance in T2 loneliness. However, this estimate may be conservative because psychological obsolescence was assessed as a broad, global construct. Future research could benefit from adopting a more domain-specific approach to identify the particular areas in which individuals experience psychological obsolescence. The present study was grounded in the theoretical assumption that perceived obsolescence threatens a sense of belonging and, consequently, contributes to loneliness. This direction was also supported by analyses, although this interpretation remains tentative given the limitation of only two measurement occasions. Nevertheless, it is also conceivable that loneliness contributes to perceived obsolescence. Lonely individuals tend to exhibit more negative self-evaluations and interpretations of social interactions (e.g., Cacioppo and Hawkley 2009), which may promote social withdrawal and a sense of being out of touch with contemporary society. In turn, this may foster perceptions of perceived obsolescence. Future research including additional measurement occasions is needed to investigate these potentially bidirectional processes more comprehensively.

## Conclusion

To conclude, using a baseline-adjusted autoregressive path model, the present study demonstrated that loneliness shows substantial temporal stability among older adults aged 75 years and older over a two-year period. Beyond this stability, psychological obsolescence was consistently associated with both baseline loneliness and follow-up loneliness after accounting for baseline loneliness and covariates. In addition, being single or divorced (compared with being partnered) and being socially isolated emerged as longitudinal predictors, whereas age, widowhood (compared with being partnered), and financial situation were associated with loneliness only in the cross-sectional analyses. Taken together, the results extend previous research by identifying psychological obsolescence as a correlate of loneliness at both baseline and follow-up, highlighting its potential importance for understanding individual differences in loneliness in later life. Further research is needed to identify the conditions under which older adults may be more vulnerable to, or protected from, perceived obsolescence.

## Supplementary Information

Below is the link to the electronic supplementary material.

Supplementary file1 (DOCX 16 KB)

## Data Availability

The data and analytic methods used in this study for replication purposes will be available upon request. The study was not pre-registered.

## Footnotes

1 Missing values for relationship status at T1 (*n* = 29) were replaced by the information provided at T2.

2 The associations between psychological obsolescence at T1 and loneliness at T1 and T2 were largely unchanged in direction and magnitude when loneliness was assessed using a single-item measure (i.e., *I feel lonely.*) rather than the 6-item scale (results not shown).
