# Supplementary material for: How perceiving pace of social change affects loneliness: psychological obsolescence as predictor for loneliness in late-life sample
Source: Eur J Ageing. 2026 Sep 17;23(1):36. doi: 10.1007/s10433-026-00951-8 (PMC13586106; doi:10.1007/s10433-026-00951-8)
Supplement: Supplementary file 1 [file 10433_2026_951_MOESM1_ESM.docx]

**Supplementary Materials**

Table of contents

**Table S1.** Cross-Lagged Panel Model Testing Bidirectionality (CLPM; *N* = 818)

**Table S2.** Baseline-Adjusted Autoregressive Model Using Complete-Case Analysis (*n* = 683)

**S1 CLPM**

To probe associations between obsolescence and loneliness, directional tests were conducted using the same covariate-adjusted specification as the primary SEM to ensure comparability across models.

Table S1. Cross-Lagged Panel Model Testing Bidirectionality (FIML; *N* = 818)

| Path | *β* | *B* | SE(*B*) | *p* |
| --- | --- | --- | --- | --- |
| Obsolescence T1 🡪 Loneliness T2 | **.075** | .202 | .079 | **.011** |
| Loneliness T1 🡪 Obsolescence T2 | .025 | .009 | .013 | .457 |
| Obsolescence T1 🡪 Obsolescence T2 | **.525** | .531 | .032 | **.000** |
| Loneliness T1 🡪 Loneliness T2 | **.601** | .602 | .030 | **.000** |

*Note.* Models additionally included covariates predicting both obsolescence at T2 and loneliness at T2: age, gender, relationship status, financial situation, subjective health, diagnoses, and social isolation; coefficients for these covariates are omitted for brevity. Descriptive statistics for obsolescence at T2 were *M* = 2.39 (*SD* = 0.96). Perceived obsolescence was positively correlated with loneliness at T2 (*r* = .25) and with obsolescence at T1 (*r* = .57). The bidirectional CLPM was fully saturated (*df* = 0) and therefore did not provide informative global fit indices.

**S2** Baseline-Adjusted Autoregressive Model Using Complete-Case Analysis (*n* = 683)

The complete-case analysis was based on 683 longitudinal respondents who provided valid responses regarding the outcome variables (i.e., loneliness) and psychological obsolescence, as well as complete data on the covariates.

Table S2. Baseline-Adjusted Autoregressive Model Using Complete-Case Analysis (*n* = 683)

| Outcome | Predictor | *β* | *B* | SE(*B*) | *p* |
| --- | --- | --- | --- | --- | --- |
| Loneliness T1 | Age T1 | -.061 | -0.039 | .023 | .090 |
|  | Gender (f=0) T1 | -.053 | -0.272 | .210 | .197 |
|  | Relationship Status (ref: partnered) T1 |  |  |  |  |
|  | Single/Divorced | **.130** | 1.019 | .285 | **.000** |
|  | Widowed | **.092** | 0.535 | .225 | **.017** |
|  | Financial Situation T1 | **-.122** | -0.368 | .109 | **.001** |
|  | ISCED (ref. low education) T1 |  |  |  |  |
|  | Middle education | -.051 | -0.260 | .264 | .323 |
|  | High education | -.056 | -0.293 | .290 | .312 |
|  | Subjective Health T1 | -.036 | -0.005 | .005 | .352 |
|  | Diagnoses T1 | .064 | 0.072 | .042 | .089 |
|  | Social Isolation T1 | **.237** | 0.797 | .119 | **.000** |
|  | Obsolescence T1 | **.235** | 0.646 | .098 | **.000** |
|  |  |  |  |  |  |
| Loneliness T2 | Loneliness T1 | **.598** | 0.590 | .031 | **.000** |
|  | Age T1 | .018 | 0.012 | .019 | .535 |
|  | Gender (f=0) T1 | -.032 | -.0162 | .172 | .346 |
|  | Relationship Status (ref: partnered) T1 |  |  |  |  |
|  | Single/Divorced | **.095** | 0.735 | .235 | **.002** |
|  | Widowed | .004 | 0.021 | .184 | .908 |
|  | Financial Situation T1 | -.025 | -0.073 | .089 | .413 |
|  | ISCED (ref. low education) T1 |  |  |  |  |
|  | Middle education | .052 | 0.262 | .215 | .223 |
|  | High education | .045 | 0.231 | .236 | .328 |
|  | Subjective Health T1 | .016 | 0.002 | .004 | .616 |
|  | Diagnoses T1 | .025 | 0.028 | .035 | .417 |
|  | Social Isolation T1 | **.073** | 0.243 | .100 | **.015** |
|  | Obsolescence T1 | **.077** | 0.209 | .082 | **.011** |

*Note.* ISCED group sizes: low education (*n* = 133), middle education (*n* = 366), high education (*n* = 295). Relationship Status group sizes: partnered/married (*n* = 481), single/divorced (*n* = 98), widowed (*n* = 238).
